# Correlation between plasma lipoprotein-associated phospholipase A_2_ and peripheral arterial disease

**DOI:** 10.3892/etm.2013.1005

**Published:** 2013-03-13

**Authors:** SHUAI-BING LI, FAN YANG, LI JING, JUAN MA, YA-DAN JIA, SHAO-YING DONG, WEI-FENG ZHENG, LUO-SHA ZHAO

**Affiliations:** Department of Cardiology, The First Affiliated Hospital of Zhengzhou University, Henan 450052, P.R. China

**Keywords:** atherosclerosis, peripheral arterial disease, lipoprotein-associated phospholipase A_2_, risk factors, biomarker

## Abstract

Lipoprotein-associated phospholipase A_2_ (Lp-PLA_2_) is a recently identified and potentially useful plasma biomarker for cardiovascular diseases. However, its role in peripheral arterial disease (PAD) remains unclear. The objective of this study was to assess the independent association of Lp-PLA_2_ and other inflammatory markers with the reduced ankle-brachial blood pressure index (ABI), a marker of PAD. We performed a cross-sectional study in 982 individuals aged ≥40 years who were recruited from the First Affiliated Hospital of Zhengzhou University. PAD was defined as an ABI <0.9 in at least one leg. The individuals were further divided into two groups, 145 with PAD and 837 without PAD. Following adjustment for traditional cardiovascular risk factors, the odds ratios of PAD when comparing the highest to the lowest quartiles were 3.24 (95% CI, 1.68–3.94) for Lp-PLA_2_, 2.14 (95% CI, 1.07–3.11) for homocysteine, 1.93 (95% CI, 1.02–4.01) for fibrinogen, 2.26 (95% CI, 1.32–5.74) for apolipoprotein B and 1.3 (95% CI, 0.75–2.49) for high-sensitivity C-reactive protein (hsCRP). When Lp-PLA_2_ and inflammatory markers were simultaneously included in the full model, the corresponding odds ratios were 1.81 (95% CI, 1.14–3.68) for Lp-PLA_2_, 1.15 (95% CI, 0.49–2.69) for homocysteine, 1.21 (95% CI, 0.88–5.57) for fibrinogen, 0.98 (95% CI, 0.51–3.85) for apolipoprotein B and 1.23 (95% CI, 1.12–3.51) for hsCRP. Lp-PLA_2_ levels were significantly and independently associated with PAD following adjustment for other inflammatory markers. These findings reflect the potential role of circulating Lp-PLA_2_ as a marker of atherosclerosis.

## Introduction

Peripheral arterial disease (PAD) is a manifestation of systemic atherosclerosis and is associated with a significantly increased risk of cardiovascular morbidity and mortality (1–3). Smoking, hypertension and diabetes mellitus are the main risk factors of PAD. Atherosclerosis is defined as an inflammatory disease (4) and previous studies have demonstrated positive associations between PAD and inflammatory markers, including high-sensitivity C-reactive protein (hsCRP), fibrinogen, homocysteine and apolipoprotein B (apo B) (5,6).

Lipoprotein-associated phospholipase A_2_ (Lp-PLA_2_) was previously characterized as a novel inflammatory biomarker correlated with atherosclerosis (7,8) that directly promotes atherogenesis (9). Lp-PLA_2_ is preferentially secreted by monocytes and macrophages and hydrolyzes oxidatively-modified low-density lipoprotein (LDL) by cleaving oxidized phosphatidylcholines, thereby generating lysophosphatidylcholine (lysoPC) and oxidized free fatty acids (10). Such chemoattractants are considered to play a pivotal role in inflammatory reactions and particularly in vascular inflammation and atherosclerosis (11). However, the potential role of Lp-PLA_2_ in atherogenesis and the anti- or pro-atherogenic characteristic of this enzyme in humans remain unclear (12). Almost all prospective and nested case cohort studies suggest that Lp-PLA_2_ is pro-atherogenic (13). However, studies on the associations between PAD and Lp-PLA_2_ in a large population are scarce.

Few studies have explored the correlation between atherosclerotic risk for peripheral arteries and mass of Lp-PLA_2_. The ankle-brachial index (ABI) is a simple non-invasive test, reflecting the ratio of the systolic blood pressure (SBP) in the ankle divided by SBP in the brachial artery. Low ABI measurements (<0.90) have been studied as a marker of atherosclerotic PAD for over 40 years (14). Low ABI measurements are sensitive and specific for flow-limiting atherosclerotic PAD (15–18). Therefore, in the present study, we measured the plasma Lp-PLA_2_ mass in a large population and investigated its correlation with anthropometric parameters and ABI to evaluate the possible contribution of Lp-PLA_2_ to PAD.

## Patients and methods

### Study population

A total of 982 individuals (487 males and 495 females, aged 40–87 years), admitted to the Cardiology Department, the First Affiliated Hospital of Zhengzhou University, China, between January 2009 and February 2012 were selected. Exclusion criteria were a cardiovascular event or vascular procedure within the preceding 6 months, elevated liver function tests (alanine transaminase >40 U/l, aspartate transaminase >40 U/l), renal disorder (serum creatinine >2.5 mg/dl) or severe congestive heart failure (New York Heart Association functional class III or IV). The details of age, gender, smoking history and medication data for use of anti-platelet drugs, anti-hypertensives drugs, anti-diabetes drugs and lipid-lowering agents were obtained through questionnaires and pill bottle reviews. The weight, height, SBP and diastolic blood pressure (DBP; with a mercury sphygmomanometer using auscultatory methods) were obtained by trained staff, with the body mass index calculated as the body weight in kilogram divided by the height in meters squared. Obesity was defined as a body mass index ≥30 kg/m^2^. Hypertension was defined as mean SBP >140 mmHg, mean DBP >90 mmHg or a self-report of a physician diagnosis or medication use. Mean blood pressure was composed of up to four readings on two separate occasions. Hypercholesterolemia was defined as a total cholesterol ≥240 mg/dl or a self-report of a physician diagnosis or medication use. Diabetes was defined as a fasting glucose ≥126 mg/dl or a nonfasting glucose ≥200 mg/dl, or a self report of a physician diagnosis or medication use. Informed consent was obtained from all participants and the study was approved by the local ethics committee.

### Laboratory measurements

Homocysteine was measured by enzyme-linked immunosorbent assay (ELISA), using reagents from Wuhan Elaborate Biotechnology Co., Ltd. (China). Fibrinogen was determined by a comparison of clotting time between patient samples and a standardized fibrinogen preparation obtained on a Coag-a-mate XC Plus automated coagulation analyzer (Organon Teknika Corp., Durham, NC, USA). Apo B was measured using standard automated enzymatic methods on a Roche Cobas Mira system (Roche Diagnostics Shanghai Ltd., Shanghai, China). hsCRP was measured using a sensitive latex particle-enhanced immunoturbidimetric assay on a Hitachi 912 automatic analyser (Hitachi, Tokyo, Japan), using reagents from Kamiya Biomedical Company (Seattle, WA, USA).

### Lp-PLA_2_ mass assay

Blood samples were collected at the baseline visit following an overnight fast and stored in aliquots frozen at −80°C. Lp-PLA_2_ mass (ng/ml) was measured using a dual enzyme linked immunoassay (Tianjin Kangerke Biological Technology Co., Ltd., China). Intra- and inter-assay coefficients of variation were <15%, respectively and sensitivity across the assay range was <0.5 ng/ml.

### Ankle-brachial index (ABI) measurements

Starting in 2009, adults aged ≥40 years were asked to participate in a lower extremity examination, including ABI, a diagnostic test for PAD with excellent performance characteristics (79–95% sensitivity and 95–100% specificity) (19). Systolic pressure was measured in the supine position in the right arm (brachial artery) and in the posterior tibial artery of the ankles using a 5 MHz Doppler probe. In all participants, blood pressures were measured twice at each site. The average of two readings was calculated. The ABI was calculated by dividing the SBP in the ankle by the SBP in the arm. We assigned a diagnosis of PAD if either leg had an ABI <0.9. Patients with ABI values >1.40 were excluded as these values may be falsely elevated due to severe vascular calcification.

### Statistical analysis

All statistical analysis was undertaken with SPSS version 15.0 (SPSS Inc., Chicago, IL, USA). Inflammatory marker data were expressed as the mean ± standard deviation. Differences between groups were analyzed by Chi-square tests. Pearson correlations between Lp-PLA_2_ and inflammatory markers were calculated. Lp-PLA_2_, homocysteine, fibrinogen, apo B and hsCRP were divided into quartiles according to the weighted distribution of the whole sample. Logistic regression was used to estimate the odds ratios for the prevalence of PAD in quartiles 2–4 of inflammatory markers compared with the first quartile. Tests for trends across increasing quartiles were computed by introducing variables with the median level for each quartile in the regression models.

Three sets of multivariable models were used to examine the association of Lp-PLA_2_, homocysteine, fibrinogen, apo B and hsCRP in a hierarchical fashion. Model 1 was adjusted for age and gender. Model 2 was further adjusted for traditional risk factors, including obesity, hypertension, hypercholesteremia, diabetes and smoking, as well as the use of medication. Model 3 adjusted simultaneously for Lp-PLA_2_, homocysteine, fibrinogen, apo B and hsCRP, in addition to the covariates included in model 2. A two-tailed P<0.05 was considered to indicate a statistically significant difference.

## Results

The clinical results of the survey of the PAD and non-PAD groups are shown in Table I. Indices in the PAD group, including homocysteine, fibrinogen, apo B, hsCRP and Lp-PLA_2_ were higher than those of the non-PAD group (all P<0.05).

Homocysteine, fibrinogen, apo B and hsCRP were strongly correlated (all r>0.50) and their correlation with Lp-PLA_2_ was extremely strong, with the exception of homocysteine (r= 0.194; Table II). Among all the inflammatory markers, Lp-PLA_2_ demonstrated the strongest correlation with apo B (r=0.938), which was similar in males and females.

In age- and gender- adjusted analyses, Lp-PLA_2_, homocysteine, fibrinogen, apo B and hsCRP levels were significantly associated with PAD (Table III, model 1). These associations persisted, although slightly attenuated, following adjustment for traditional cardiovascular risk factors. In the risk factor adjusted models, the odds ratios comparing the prevalence of PAD in the highest vs. the lowest quartiles were 3.24 (95% CI, 1.68–3.94) for Lp-PLA_2_, 2.14 (95% CI, 1.07–3.11) for homocysteine, 1.93 (95% CI, 1.02–4.01) for fibrinogen, 2.26 (95% CI, 1.32–5.74) for apo B and 1.37 (95% CI, 0.75–2.49) for hsCRP (Table III, model 2).

When Lp-PLA_2_, homocysteine, fibrinogen, apo B and hsCRP were introduced in the models, in addition to traditional cardiovascular risk factors, Lp-PLA_2_, apo B and fibrinogen were still associated with PAD prevalence (Table III, model 3). The odds ratios comparing the prevalence of PAD in the highest vs. the lowest quartiles were 1.81 (95% CI, 1.14–3.68) for Lp-PLA_2_, 1.15 (95% CI, 0.49–2.69) for homocysteine, 1.21 (95% CI, 0.88–5.57) for fibrinogen, 0.98 (0.51–3.85) for apo B and 1.23 (95% CI, 1.12–3.51) for hsCRP. In subgroup analyses, the association of Lp-PLA_2_ with PAD was similar irrespective of the presence or absence of traditional risk factors and other inflammatory markers (not shown).

## Discussion

The salient observations of the present study are that plasma levels of Lp-PLA_2_ are elevated in patients with PAD and are independent of homocysteine, fibrinogen, apo B and hsCRP. Lp-PLA_2_ independently affected the presence of PAD following adjustment for traditional risk factors.

Lp-PLA_2_ is a strong independent and novel inflammatory biomarker for cardiovascular events (20) and the prevalence and progression of subclinical atherosclerosis (21). Previous evidence suggests that inflammation is an important pathogenic factor in atherosclerosis and CHD. Furthermore, atherosclerosis is recognized as a manifestation of vascular inflammation (4). Inflammation is associated with almost all stages of vascular disease, including atherogenesis, plaque rupture and end-organ damage secondary to ischemia and/or embolism. The results of previous studies indicate that Lp-PLA_2_ mass plays a key role in the evolution of atherosclerosis through various mechanisms leading to initiation, propagation and subsequent complications of atherosclerotic plaque formation (7,8). Lp-PLA_2_, originally named platelet-activating factor acetylhydrolase, is an enzyme involved in lipoprotein metabolism and inflammatory pathways (10). In our study, apo B may contribute to Lp-PLA_2_ mass changes. An increase in plasma Lp-PLA_2_ mass, reflecting lipoprotein particles, has been established in several investigations (22,23). Stafforini *et al* indicated that Lp-PLA_2_ participates in the key oxidative steps of atherogenesis due to the association of Lp-PLA_2_ and LDL via an interaction with apo B (24). In humans, 80% of Lp-PLA_2_ circulates bound to LDL, 10–15% circulates with high-density lipoprotein and the remaining 5–10% circulates with very low-density lipoprotein (VLDL) (25). Lp-PLA_2_ enzymatic activity results in the generation of lysoPC and oxidized nonesterified fatty acids, two pro-inflammatory mediators (10). LysoPC stimulates macrophage proliferation, upregulates cytokines and CD40 ligands and increases the expression of vascular adhesion molecules, implying a complex interaction between Lp-PLA_2_ and other inflammatory mediators (26,27). Confirmation of these findings in prospective studies is of critical importance.

In our study, fibrinogen and apo B were independently associated with the prevalence of PAD after taking into account traditional cardiovascular risk factors, use of medications and all other inflammatory markers. This finding is consistent with previous reports demonstrating an association between fibrinogen and incident coronary heart disease (CHD) events, as well as subclinical atherosclerosis adjusting for other inflammatory markers (28). An association between baseline fibrinogen levels and PAD was observed in a cross-sectional study of 3,949 individuals in the 1999–2002 National Health and Nutrition Examination Survey (NHANES) (29). Higher fibrinogen levels may potentially promote atherosclerosis by increasing platelet adhesion to the subendothelium, as well as by affecting endothelial function (5). Our findings support the role of fibrinogen as an independent marker of generalized atherosclerotic lesions in major arterial beds.

To the best of our knowledge, the present study is the first to demonstrate a cross-sectional association between Lp-PLA_2_ and the presence of PAD. Thus, Lp-PLA_2_ appears to be a marker that may have clinical utility in assessing the risk of developing PAD. In conclusion, this study demonstrates for the first time an independent association between Lp-PLA_2_ and PAD, defined as a reduced ABI, in a large population based study. More detailed characterization of the association between Lp-PLA_2_ and clinical and subclinical atherosclerotic outcomes is required to better characterize the role of inflammatory cells in atherosclerosis.

## Figures and Tables

**Table I:** Clinical characteristics of PAD and non-PAD groups.

Characteristics	PAD group	non-PAD group	P-value
N	145	837	
Age (years)	62.67±11.12	61.27±10.59	0.017
Gender (% male)	47.5	30	0.048
BMI ≥30 kg/m^2^	38	30	0.490
Hypertension (%)	58	47	0.025
Hypercholesteremia (%)	51	46	0.209
Diabetes (%)	18	11	0.036
Smoking (%)	47.2	32.8	0.042
Inflammatory markers			
HCY (*μ*mol/l)	15.89±4.99	15.47±5.32	0.008
FIB (g/l)	3.57±1.20	2.89±0.68	0.021
Lipoprotein(a) (g/l)	0.80±0.41	0.37±0.21	0.011
hsCRP (mg/l)	22.23±11.74	11.20±5.89	0.031
Lp-PLA_2_ (ng/ml)	560.33±201.91	307.94±134.31	0.001
Medications			
Anti-platelet drugs (%)	88.6	31.2	0.231
Anti-hypertensive drugs (%)	28.8	20.1	0.306
Anti-diabetes drugs (%)	7.7	7.2	0.135
Lipid-lowering agents (%)	30.6	35.2	0.041

PAD, peripheral arterial disease; BMI, body mass index; HCY, homocysteine; FIB, fibrinogen; hsCRP, high-sensitivity C-reactive protein; Lp-PLA_2_, lipoprotein-associated phospholipase A_2_.

**Table II:** Correlation coefficients between inflammatory markers.

	Lp-PLA_2_	Homocysteine	Fibrinogen	Apo B
Lp-PLA_2_	-	-	-	-
Homocysteine	0.194	-	-	-
Fibrinogen	0.538	0.505	-	-
Apo B	0.938	0.577	0.714	-
hsCRP	0.891	0.626	0.557	0.661

Lp-PLA_2_, lipoprotein-associated phospholipase A_2_; apo B, apolipoprotein B; hsCRP, high-sensitivity C-reactive protein. All were P<0.05.

**Table III:** Odds ratios of peripheral arterial disease by inflammatory markers.

Model	Quartile 2	P-value	Quartile 3	P-value	Quartile 4	P-value
Lp-PLA_2_						
1	1.26 (0.53–3.02)	0.81	2.26 (1.97–4.27)	0.37	3.49 (1.32–3.70)	0.02
2	0.89 (0.32–3.65)	0.54	1.98 (1.21–4.14)	0.21	3.24 (1.68–3.94)	0.03
3	0.63 (0.57–2.39)	0.25	1.34 (0.72–3.11)	0.04	1.81 (1.14–3.68)	0.01
Homocysteine						
1	2.44 (1.73–3.92)	0.57	3.22 (2.46–4.79)	0.47	3.49 (1.32–3.70)	0.06
2	1.87 (0.82–2.65)	0.44	2.28 (1.61–5.24)	0.37	2.14 (1.07–3.11)	0.04
3	1.03 (1.14–3.76)	0.35	1.17 (2.12–4.55)	0.28	1.15 (0.49–2.69)	0.15
Fibrinogen						
1	0.89 (0.87–2.90)	0.15	2.34 (1.77–4.40)	0.23	2.35 (1.52–5.29)	0.03
2	0.65 (0.62–2.73)	0.48	1.97 (0.63–3.87)	0.20	1.93 (1.02–4.01)	0.03
3	0.50 (0.36–1.42)	0.39	1.42 (0.82–3.37)	0.06	1.21 (0.88–5.57)	0.04
Apolipoprotein B						
1	0.75 (0.28–2.27)	0. 34	0.79 (0.26–2.15)	0.57	1.26 (0.08–0.81)	0.02
2	0.66 (0.19–2.30)	0.35	0.59 (0.19–2.41)	0.08	2.26 (1.32–5.74)	0.01
3	0.57 (0.22–2.70)	0.63	0.83 (0.06–0.86)	0.24	0.98 (0.51–3.85)	0.01
hsCRP						
1	0.79 (0.28–2.27)	0.34	1.26 (0.53–3.00)	0.06	1.54 (0.62–3.80)	0.03
2	0.75 (0.26–2.15)	0.25	0.94 (0.22–3.94)	0.13	1.37 (0.75–2.49)	0.04
3	0.66 (0.19–2.30)	0.12	0.87 (0.21–3.59)	0.09	1.23 (1.12–3.51)	0.20

Data are presented as aOR (95% CI). aOR, adjusted odds ratio; CI, confidence interval; hsCRP, high-sensitivity C-reactive protein; Lp-PLA_2_, lipoprotein-associated phospholipase A_2_. Model 1, adjusted for age and gender; model 2, further adjusted for smoking status, diabetes, hyper-tension, high cholesterol, use of medication and body mass index (normal, overweight or obese); model 3, further adjusted for all other inflammatory markers. Quartile 1 used as a control group (not shown in table).
